# Rupture de l’aorte thoracique descendante suite à une décélération brutale post traumatique

**DOI:** 10.11604/pamj.2020.35.17.15777

**Published:** 2020-01-23

**Authors:** Soufiane Badsi, Abderrahim El kaouini, Soufiane Diyas, Brahim Housni

**Affiliations:** 1 Service de Réanimation, CHU Mohamed VI, Faculté de Médecine et de Pharmacie d’Oujda, Université Mohammed 1^er^ Oujda, Oujda, Maroc

**Keywords:** Accident voie publique, rupture post traumatique, aorte descendante, traitement chirurgical, Road traffic accident, post traumatic rupture, descending aorta, surgical treatment

## Abstract

La rupture aigue post traumatique de l’aorte thoracique constitue la seconde cause de mortalité chez les accidentés de la route; 80% des patients décèdent sur les lieux de l’accident. L’atteinte de sa portion descendante en dehors de l’isthme est rare et évaluée à moins de 3%. Nous rapportons le cas d’un homme de 60 ans, victime d’un accident de la voie publique (AVP) dont le mécanisme était un motocycliste heurté par une voiture avec points d’impacts au niveau du membre supérieur droit et thoracique. Cet AVP a occasionné une fracture des deux os de l’avant bras et une rupture de l’aorte thoracique descendante (ATD). Le traitement a consisté en premier temps à une suture de la brèche aortique complétée par la mise en place d’une prothèse synthétique circonférentielle avec anastomose aorto-aortique et en 2^ème^ temps à la mise en place d’une plaque vissée de l’avant bras. L’évolution était bonne et le malade fut transféré au Service de Chirurgie Vasculaire.

## Abstract (en)

Acute post-traumatic rupture of the thoracic aorta is the second cause of road traffic deaths; 80% of patients die at the scene of the accident. The involvement of its descending portion outside the isthmus is rare and it is estimated to be less than 3%. We report the case of a 60-year old man, victim of road traffic accidents (RTA) whose mechanism was: the motorcyclist was struck by a car; the points of impact included the right upper limb and the chest. This RTA caused a fracture in both bones of the forearm and a rupture of the descending thoracic aorta (ATD). Treatment was based in the first time on the suture of the aortic rupture complemented by synthetic circumferential prosthesis implantation with aorto-aortic anastomosis and in a second time on the application of a screwed plate in the forearm. The outcome was good and the patient was referred to the Department of vascular surgery.

## Introduction

La rupture aiguë post traumatique de l’aorte thoracique constitue la seconde cause de mortalité chez les accidentés de la route; 80% des patients décèdent sur les lieux de l’accident [1]. L’atteinte de sa portion descendante en dehors de l’isthme est rare et évaluée à moins de 3% [2]. Elle constitue donc une véritable urgence médicochirurgicale dont le diagnostic précoce et la prise en charge initiale rapide améliore nettement le pronostic vital des malades.

## Patient et observation

Nous rapportons le cas d’un homme de 60 ans, victime d’un AVP dont le mécanisme était un motocycliste heurté par une voiture avec points d’impacts au niveau du membre supérieur droit et thoracique. Cet AVP a occasionné une fracture des deux os de l’avant bras et une rupture de l’aorte thoracique descendante (ATD). L’examen général était sans particularités. L’examen locomoteur a objectivé un avant bras droit déformé. Le bilan lésionnel initial (scanner corps entier) a permis de mettre en évidence une dissection de l’aorte thoracique descendante de 42 x 31 mm. Les lésions associées comprenaient un hémothorax bilatéral de faible abondance et une fracture des deux os de l’avant bras. Le traitement a consisté en premier temps à une suture de la brèche aortique complétée par la mise en place d’une prothèse synthétique circonférentielle avec anastomose aorto-aortique et en 2^ème^ temps à la mise en place d’une plaque vissée de l’avant bras. L’évolution était bonne et le malade fut transféré au Service de Chirurgie Vasculaire.

## Discussion

Les lésions post traumatiques de l’ATD sont rares. Les accidents de la voie publique demeurent la principale cause avec un taux élevé de mortalité avoisinant les 80 à 85% sur les lieux d’accidents ou pendant le transport aux urgences [3]. Les mécanismes des lésions traumatiques de l’aorte thoracique concernent les traumatismes à haute cinétique et sont principalement indirecte liés à la décélération: comme c’était le cas de notre patient; ou à l’accélération, principal mécanisme des lésions traumatiques qui agit par cisaillement de la paroi, entraînant la lacération de celle-ci au niveau des zones de transition entre une aorte mobile et une zone où l’aorte est fixe ou plus rigide [4]. Cliniquement, le syndrome de pseudocoarctation traumatique semble fréquent dans les lésions de l’ATD. Il comporte: différence entre pressions artérielles aux membres supérieurs et inférieurs, asymétrie des pouls périphériques et signes périphériques d’ischémie. Notre malade était asymptomatique.

Le diagnostic de la dissection de l’aorte thoracique repose essentiellement sur la réalisation de la tomodensitométrie (TDM) et l’échocardiographie transœsophagienne (ETO) qui sont équivalentes en termes de sensibilité et de spécificité [5]. En pratique courante, le scanner thoracique constitue la pierre angulaire du diagnostic. Notre malade a bénéficié d’un scanner corps entier dans le cadre du bilan lésionnel post traumatique. Le développement de la radiologie interventionnelle a étendu le champ des méthodes thérapeutiques endovasculaires avec recours aux endoprothèses aortiques dans le contexte de la pathologie aortique aiguë post traumatique. Toutes les études rapportées ces dernières années insistent sur la sûreté et l’efficacité du traitement endovasculaire de la rupture de l’aorte descendante avec réduction de la morbidité et de la mortalité de ces patients, par rapport à ceux ayant bénéficié d’un traitement chirurgical conventionnel [6, 7]. En effet, l’utilisation d’endoprothèses représente une alternative moins invasive à la chirurgie réparatrice, la thoracotomie est évitée, de même que le clampage aortique et la circulation extracorporelle qui sont délétères pour les patients. Du fait du manque de moyen dans notre structure, le malade a bénéficié d’un traitement chirugical. Le traumatisme de l’avant bras, a également été opéré avec des suites opératoires simples.

## Conclusion

Les lésions traumatiques de l’aorte thoracique descendante sont moins connues des anesthésistes réanimateurs que celles de l’isthme aortique car elles sont rares. Elles doivent être recherchées de manière systématique devant tout traumatisme thoracique. Le diagnostic et le choix du moment de prise en charge ont été améliorés par les progrès de la radiologie et des différentes techniques thérapeutiques.
